# Editorial: Spatial single-cell sequencing in studying solid cancer development

**DOI:** 10.3389/fimmu.2023.1342989

**Published:** 2023-12-12

**Authors:** Behzad Mansoori, Nicola Silvestris, Behzad Baradaran

**Affiliations:** ^1^ Molecular and Cellular Oncogenesis Program, The Wistar Institute, Philadelphia, PA, United States; ^2^ Department of Human Pathology in Adulthood and Childhood Gaetano Barresi, University of Messina, Messina, Italy; ^3^ Immunology Research Center, Tabriz University of Medical Sciences, Tabriz, Iran

**Keywords:** heterogeneity, single cell sequencing (scRNA-seq), intratumoral, temporal, intertumoral, epigenetic, spatial

Solid cancers, characterized by their multifaceted nature, present formidable challenges in therapeutic interventions. Central to these challenges is the inherent heterogeneity of tumors and the suppressive dynamics of the tumor microenvironment (TME). The TME, a complex milieu comprising immune cells, blood vessels, and other stromal elements, not only interacts with cancer cells but also significantly influences tumor progression and resistance mechanisms. This interaction is further complicated by the diverse composition of cell types within tumors, each bearing distinct molecular signatures. Tumor heterogeneity, both genetic and non-genetic, emerges from a myriad of factors, including genomic instability, epigenetic modifications, and variances in the microenvironment. Current therapeutic paradigms, which often perceive cancer as a uniform disease, are ill-equipped to address this diversity, leading to potential drug resistance. It is, therefore, imperative to recognize and understand the intricate interplay between various tumor subtypes, immune cells, and other TME components. A profound comprehension of these complexities, as underscored by recent scientific literature, is pivotal for the advancement of diagnostic tools and the formulation of tailored therapies. These strategies, designed to target the myriad of cancer cell populations within tumors, hold the promise of more effective and personalized therapeutic interventions.

Tumor heterogeneity can be understood in multiple dimensions:

1. Intertumor Heterogeneity refers to the variability between tumors from different patients. Even if two individuals have the same type of cancer, their tumors might exhibit distinct genetic profiles.
2. Intratumor Heterogeneity denotes the genetic and molecular variations within different regions of a single tumor. This internal diversity complicates treatment strategies, as one part of the tumor might respond differently than another.
3. Temporal Heterogeneity captures the evolutionary nature of tumors, where their genetic makeup can change over time, potentially rendering initial treatments ineffective at later stages.
4. Epigenetic Heterogeneity involves changes that affect gene expression without altering the DNA sequence, such as DNA methylation.
5. Spatial Heterogeneity highlights the genetic discrepancies in different spatial regions of a tumor, influenced by factors like varying oxygen and nutrient levels.

Accurate analysis of tumor heterogeneity is crucial for effective cancer treatment. Single-cell sequencing technologies have revolutionized the way tumor heterogeneity is characterized. Single-cell DNA sequencing technologies excel in detecting rare clones, reconstructing clonal structures, and pinpointing both simultaneous and distinct mutations within individual cells. On the other hand, single-cell RNA sequencing (scRNA-seq) has greatly advanced the study of functional heterogeneity, enhancing our understanding of the molecular dynamics of cancer progression. This technology enables detailed classification of cell types in complex samples from primary tumors. The latest single-cell transcriptome atlases, which include both healthy and diseased samples from humans and mice, facilitate this detailed analysis. This comprehensive approach naturally leads to a pivotal question: How can single-cell technologies help us to understand tumor heterogeneity more profoundly? By delving into the genetic and molecular landscape of tumors at the cellular level, these technologies have revolutionized cancer research, offering unprecedented insights and opening new avenues for targeted and effective cancer treatments.

## 1 Intertumoral heterogeneity

scRNA-seq provides detailed biological information at the cellular level, enabling the identification of distinct genetic profiles in tumors from different patients. Unlike traditional ‘bulk’ RNA-sequencing, which averages out the differences across all cells in a sample, scRNA-seq analyzes individual cells. This allows for a much more detailed and nuanced understanding of the biological characteristics at the cellular level.

## 2 Intratumor heterogeneity

scRNA-seq allows for the profiling of genetic and molecular variations within different regions of a single tumor. Patel et al. utilized RNA sequencing (RNA-seq) to uncover intratumor heterogeneity in glioblastoma for this purpose they first, they profiled individual cells within tumors, revealing diverse gene expression patterns that highlight heterogeneity. They also detected large-scale copy number variations (CNVs) within tumor cells, indicative of genetic diversity. Techniques like clustering and multidimensional scaling were used to analyze RNA-seq data, grouping cells based on their expression profiles and further illustrating variability within the tumor. The study included RNA-seq data from normal brain tissue, allowing for the distinction between normal and malignant cells and emphasizing tumor heterogeneity. Additionally, a range of stemness-related expression states in tumor cells was identified, indicating variability in their potential for self-renewal and differentiation. Finally, they observed that glioblastoma subtype classifiers vary across individual tumor cells, suggesting heterogeneity in tumor subtypes.

## 3 Temporal heterogeneity

The evolutionary nature of tumors, where their genetic makeup changes over time. This can be identified by linking temporally matched cDNA-seq and scRNA-seq data. Zhang et al. utilized RNA sequencing (RNA-seq) to identify temporal heterogeneity in cell differentiation processes. They performed single-cell RNA-seq (scRNA-seq) analysis at various time points during neural differentiation from mouse embryonic stem cells, using cell population RNA-seq (cpRNA-seq) as a reference to guide time point selection and examine intercellular heterogeneity. Through saturation analysis, they determined the sufficiency of scRNA-seq samples in capturing major intercellular heterogeneity at each differentiation stage. They developed a computational model using cpRNA-seq data to infer differentiation time and applied it to scRNA-seq data, allowing for the unbiased association of cell-cycle checkpoints with the internal molecular timer of single cells. Additionally, they inferred regulatory networks accounting for intercellular heterogeneity by linking cpRNA-seq and scRNA-seq data, identifying key regulatory genes involved in cell differentiation timing. The study further validated the roles of these regulatory genes in controlling cell cycle and differentiation timing through gene knockout experiments. This approach is vital for understanding how tumors evolve, potentially impacting the effectiveness of initial treatments at later stages.

## 4 Epigenetic heterogeneity

While scRNA-seq primarily focuses on gene expression, it can also integrated with epigenetic study. Khouri-Farah et al. integrated scRNA-seq with single-nucleus ATAC sequencing (snATAC-seq) in their epigenetic study to show how these advanced techniques can be used to unravel the complexities of cell state transitions and lineage commitments, particularly in the embryonic mouse cerebellum. This integration is pivotal in identifying and understanding the epigenetic heterogeneity within cellular populations, a concept that can be extended to tumor analysis. By analyzing both the gene expression profiles and the accessible chromatin states at the single-cell level, the study provides a comprehensive view of the regulatory mechanisms governing cell fate. This approach not only highlights the dynamic interplay between transcriptional changes and epigenetic modifications but also sets a precedent for exploring similar complexities in tumor cells, where understanding heterogeneity is crucial for developing targeted therapies and comprehending tumor evolution and resistance to treatments.

## 5 Spatial Heterogeneity

scRNA-seq can highlight genetic discrepancies in different spatial regions of a tumor. This is particularly important in understanding how various factors, such as oxygen and nutrient levels, influence the genetic landscape of different tumor regions. Davide Risso et al. highlight the capabilities of scRNA-seq in elucidating spatial heterogeneity in biological tissues. This technique allows for the detailed characterization of individual cells’ molecular states, providing insights into the diverse transcriptional profiles present across different spatial regions. By enabling the assessment of cell-to-cell variability in gene expression, scRNA-seq plays a crucial role in disentangling the complexity of heterogeneous tissues. It helps identify distinct cell types and states in various spatial regions, thereby shedding light on the spatial organization of tissues. Additionally, scRNA-seq reveals dynamic interactions and signaling pathways that contribute to spatial heterogeneity, enhancing our understanding of how cells influence each other within their spatial context. Technological advancements, such as the zero-inflated negative binomial model (ZINB-WaVE), further refine the analysis of scRNA-seq data, improving the accuracy of interpretations regarding spatially heterogeneous samples.

Through this Research Topic, we have explored the multifaceted challenges posed by solid cancers, highlighting the pivotal role of tumor heterogeneity in shaping therapeutic interventions. The advent of single-cell sequencing technologies, notably scRNA-seq, has marked a revolution in our ability to characterize this heterogeneity. These advanced techniques have unveiled intricate details of the genetic and molecular landscape of tumors at the cellular level, allowing us to discern distinct genetic profiles within individual tumors. However, to gain a more profound understanding of tumor heterogeneity, it’s crucial to extend our focus to single-cell multi-omics. This approach combines genomic, transcriptomic, proteomic, and metabolomic data at the single-cell level, offering a more comprehensive view of the cancer cells’ state and behavior. This is not just an advancement in technology; it represents a paradigm shift in how we understand and treat cancer.

## Author contributions

BM: Writing – original draft, Writing – review & editing. NS: Writing – review & editing. BB: Writing – review & editing.

